# Treatment of Port Wine Birthmark With Nude Tattoo Using Multiple Laser Modalities

**DOI:** 10.1111/jocd.70178

**Published:** 2025-04-08

**Authors:** Vincent Pecora, Emily Murphy, Pooja Sodha

**Affiliations:** ^1^ Department of Dermatology The George Washington University School of Medicine and Health Sciences Washington DC USA

**Keywords:** cosmetic dermatology, laser therapy, multimodal laser, port wine birthmark, PWB

## 1 Introduction

Port wine birthmarks (PWB) are congenital capillary malformations that frequently occur on the face and neck [1, 2]. Activating mutations in GNAQ and PI3K, along with other predisposing mutations in PWB, lead to dysregulated MAPK and PI3K signaling, triggering vascular ectasia and dilation [2]. PWB are initially flat red macules or patches, but they progressively darken, undergo soft tissue hypertrophy, and develop vascular nodules into late childhood and adulthood [2]. PWB are important to recognize given their cosmetic impact, especially on the face, and possible associations with genetic syndromes, like Sturge–Weber. First‐line treatments of PWB include the pulsed dye laser (PDL), which targets and destroys hemoglobin, causing necrosis of vessel walls [2, 3]. One alternative treatment is medical tattooing to alter the skin color and camouflage the PWB [4].

However, the use of pulsed dye laser therapy to treat cutaneous pathologies with tattoo overlays has remained controversial. High energy, short‐pulsed dye lasers have specifically been found to increase the risk of irreversibly darkening cosmetic medical tattoos, leading to gray‐black hyperpigmentation [4]. In order to prevent adverse cosmetic outcomes in patients with pre‐existing tattoos, recent therapies have utilized multimodal pulsed‐dye laser therapy to selectively target dilated blood vessels and reduce diffuse erythema without destroying surrounding tissue [5]. To date, there are no studies utilizing more than two simultaneous forms of multimodal laser therapy in the treatment of PWB. Here, we present a patient with a nude tattoo over a facial PWB that was successfully treated with multiple treatments of multimodal long‐pulsed 755 nm Alexandrite, 595 nm pulsed dye laser, and 10 600 nm fractional carbon dioxide (CO_2_) laser therapy on the same day. These results demonstrate that the presence of medical tattoos should not deter physicians from utilizing multimodal laser therapy to treat PWB.

## 2 Case Report

A 64‐year‐old female presented with a facial PWB on the left temple, cheek, nose, and cutaneous upper lip. Six rounds of multimodal laser therapy were utilized, consisting of long‐pulsed 755 nm Alexandrite (Lutronic Clarity) (Table 1) and long‐pulsed 595 nm (Candela Vbeam) PDL (Table 2) with the addition of a 10 600 nm fractional CO_2_ laser (Candela Co2RE or DEKA DOT) laser three times (lasers were applied in this order). The treatments took place over 22 months.

**TABLE 1:** Treatment of tattooed facial PWB with long‐pulsed 755 nm Alexandrite (Lutronic Clarity).

Session	Fluence (J/cm2)	Pulse duration (ms)	Spot size (mm)	Cryogen	Number of spots
1	22	3.0	15	30‐20‐10	43
2	22 (temples); 24 (left cheek); 20 (upper lip)	3.0	15	30‐20‐10	33
3^a^	22 (temples); 20 (left cheek)	3.0	15	30‐20‐10	38
4	22	3.0	15	30‐20‐10	22
5^b^	24; 20 (left cheek, upper lip)	3.0	15	30‐20‐10	21
6^c^	24	3.0	15	30‐20‐10	24

^a^ Denotes first treatment with administration of 10 600 nm fractional CO_2_RE laser (Candela Co2re, 55 mJ, 311 J/cm^2^).

^b^ Denotes second administration with 10 600 nm fractional CO_2_RE laser (Co2RE, 50 mJ, 283 J/cm^2^).

^c^ Denotes third treatment with 10 600 nm fractional CO_2_ laser (Deka DOT, 25 W, 1500 dwell time, 750 spacing, 1 pass with 1–2 stacked pulses). Cold ice packs and forced cold air were utilized after each treatment.

**TABLE 2:** Treatment of tattooed facial PWB with 595 nm PDL (Candela Vbeam).

Session	Fluence (J/cm^2^)	Pulse duration (ms)	Spot size (mm)	Cryogen	Number of spots
1	7.5	3.0	10	30/30	382
2	7.75 (temple, lower cheek, glabella); 7.0 (cheek)	1.5	10	30/30	68
3^a^	7.75 (temples); 7.25 (left cheek); 6.5 (right cheek); 6.25 (nose); 1.5 (temple, left cheek); 3 (right cheek, nose)	1.5 (temple, left cheek); 3 (right cheek, nose)	10	30/30	111
4	8 (left cheek); 7.5 (upper cutaneous lip)	1.5	10	30/30	139
5^b^	7.75 (temple, cheek, upper lip); 6.25 (left cheek, nose)	1.5 (temple, left cheek); 3 (right cheek, nose)	10	30/30	122
6^c^	8	3	10	30/30	126

^a^ Denotes first treatment with 10 600 nm fractional CO_2_ laser (Candela Co2RE, 55 mJ, 311 J/cm^2^).

^b^ Denotes second administration with 10 600 nm fractional CO_2_ laser (Co2RE, 50 mJ, 283 J/cm^2^).

^c^ Denotes third treatment with 10 600 nm fractional CO_2_ laser (Deka DOT, 25 W, 1500 dwell time, 750 spacing, 1 pass with 1–2 stacked pulses). Cold ice packs and forced cold air were utilized after each treatment.

**FIGURE 1:**
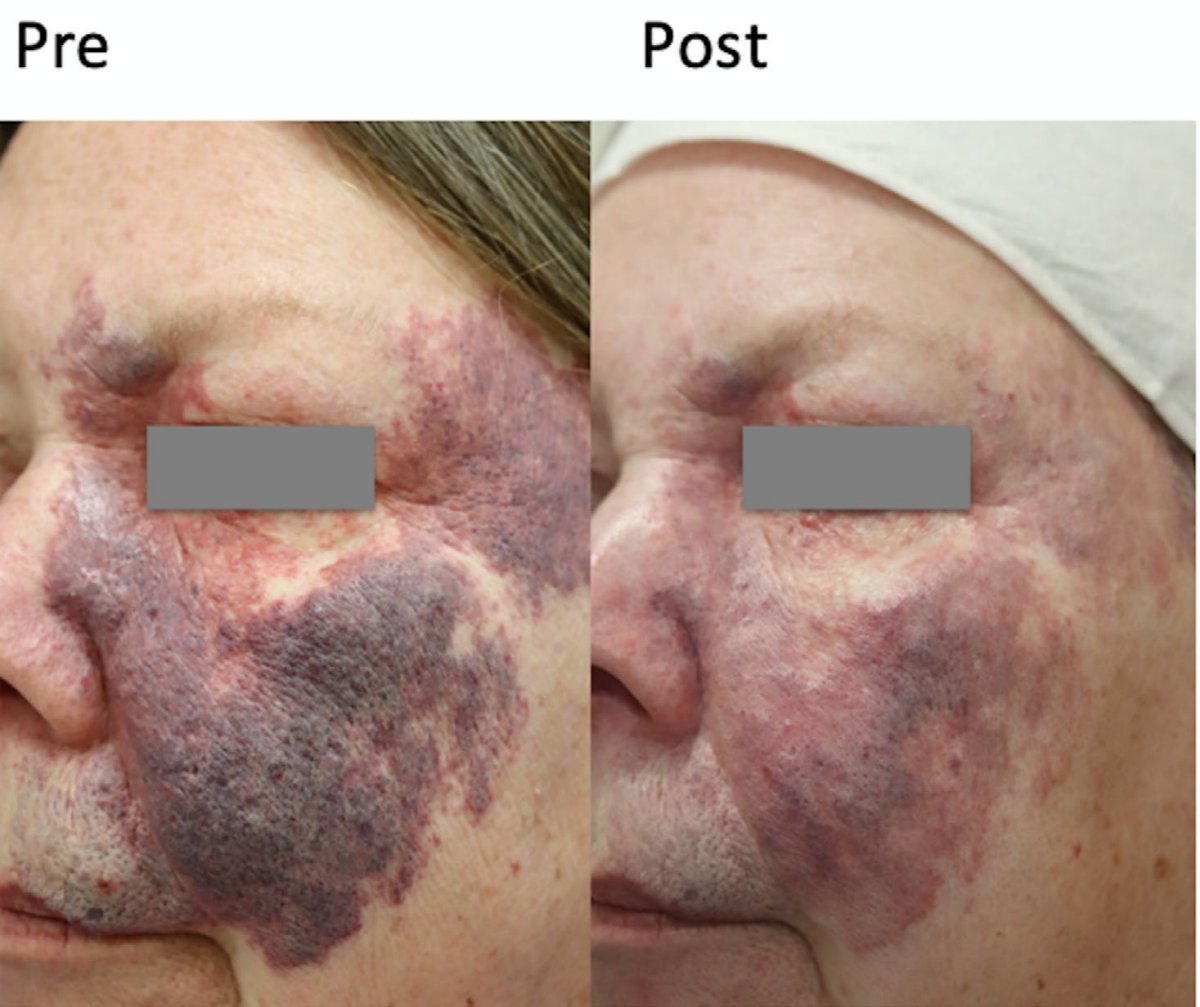
Pre‐ and Post‐Treatment reduction of facial PWS with red facial tattoo overlay. A total of five treatments were given with the first two given 8 months apart and the third, fourth, and fifth given 1 month apart.

During the third, fifth, and sixth treatment sessions, a 10 600 nm fractional CO_2_ laser was applied to the entire lesion in addition to the 755 nm Alexandrite and 595 nm PDL. The endpoint for the Alexandrite laser, which was applied to the purple hypertrophic tissue only, was a slow faint bluish darkening of the PWB, and the endpoint for the pulsed dye laser, applied to the entire lesion, was sustained purpura. The number of long‐pulsed 755 nm Alexandrite (Clarity) pulses required for each treatment progressively reduced from 43 to 21 over five treatments, thereby marking a progressive reduction in the texture and vascular density of the PWB. Between each visit, the patient reported further clearing of the PWB, particularly in the temple. A small blister was noted by the patient after the second treatment along the left upper cheek, which resolved with a small scar noted at her third treatment. Subsequent use of the CO_2_ fractional laser improved the scar with no appreciable sequelae after the last treatment (Figure 1).

## 3 Discussion

Laser therapy is the first‐line treatment for PWB as photons target capillaries and postcapillary venules to destroy endothelial lining without disrupting nearby tissues [3]. The use of laser therapy to treat PWB with superimposed medical tattoos is more challenging due to concerns about darkening pre‐existing tattoo pigments leading to worsened cosmesis cosmetic appearance [5]. However, recent studies have found that utilizing multimodal laser therapy can selectively target dilated blood vessels without damaging surrounding tissue, thereby reducing the likelihood of adverse cosmetic outcomes [5, 6].

Irreversible tattoo pigment darkening has been described in multiple cases since first being present in 1993 [5]. In some instances, further laser treatments can improve the pigmentation, and in other cases, other laser modalities must be employed. This unique case presented both the chronicity of an untreated PWB with hypertrophy and increased vascular density along with overlaid nude cosmetic tattoo. To date, there are currently no studies that have utilized a combination of three different laser types on the same day in the treatment of PWB. Here, we show that the use of a multimodal laser therapy successfully improved all features, along with the added improvement of upper lip conformation/lift. The combination of long‐pulsed 755 nm Alexandrite (Clarity), long‐pulsed 595 nm PDL, and 10 600 nm fractional CO_2_ laser induced significant PWB clearance by targeting both tattoo pigment, capillary vessels, and reactive tissue hypertrophy of the PWB. While the 595 nm PDL laser likely played a role in selectively targeting the pigment of this patient's facial tattoo, the addition of the longer wavelength of the deeper penetrating Alexandrite laser and the coagulative effect of the fractional CO_2_ laser improved the darker violaceous hue of the underlying PWB. This highlights the importance of continued therapy or therapy initiation even for adult patients with a more complicated presentation. The use of simultaneous, triple multimodal laser therapy on the same day is therefore highly effective in reducing the pigmentation of chronic PWB with hypertrophy and vascular density from pre‐existing tattoo pigments. Slow titration of use with these additional devices is important as excessive heat around sensitive structures may induce scarring.

## 4 Conclusion

No studies to date have employed a multimodal therapy regimen with greater than two laser types in the treatment of PWB. While the use of laser therapy to treat PWB with pre‐existing tattoos has been controversial, we present a patient with a nude tattoo overlying a facial PWB that was successfully treated using multimodal laser therapy consisting of long‐pulsed 755 nm Alexandrite laser, 595 nm pulsed dye laser, and 10 600 nm fractional CO_2_ laser.

## Ethics Statement

The authors confirm that the ethical policies of the journal, as noted on the journal's author guidelines page, have been adhered to. No ethical approval was required as this is a review article with no original research data.

## Consent

A photo consent form was completed by the patient in this case report and was included in the submission materials.

## Conflicts of Interest

The authors declare no conflicts of interest.

## Data Availability

The data that support the findings of this study are available from the corresponding author upon reasonable request.
